# Skene duct adenocarcinoma in a patient with an elevated serum prostate-specific antigen level: a case report

**DOI:** 10.1186/s13256-017-1558-y

**Published:** 2018-02-14

**Authors:** Sohgo Tsutsumi, Takashi Kawahara, Yusuke Hattori, Taku Mochizuki, Jun-ichi Teranishi, Kazuhide Makiyama, Yasuhide Miyoshi, Masako Otani, Hiroji Uemura

**Affiliations:** 1 Departments of Urology and Renal Transplantation, Yokohama City University Medical Center, Yokohama, Japan; 2 Department of Urology, Yokohama City University Graduate School of Medicine, Yokohama, Japan; 3 Division of Diagnostic Pathology, Yokohama City University Medical Center, Yokohama, Japan

**Keywords:** Prostate-specific antigen, Female genital neoplasm, Skene adenocarcinoma, Urethral adenocarcinoma, Urethral carcinoma

## Abstract

**Background:**

Female urethral carcinoma is a very rare disease that accounts for 0.02% of malignant diseases in female patients.

**Case presentation:**

A 70-year-old Asian Japanese woman with a urethral tumor was referred to our hospital to undergo further examination. Biopsy specimens showed urethral adenocarcinoma that was positive for prostate-specific antigen. Her serum prostate-specific antigen level before surgery was 34.4 ng/ml. Urethral tumor resection with pelvic lymph node resection was performed. Her serum prostate-specific antigen level decreased to < 0.01 ng/ml after surgery.

**Conclusions:**

We report a very rare case of Skene duct adenocarcinoma in a female patient with serum prostate-specific antigen elevation.

## Background

Female urethral carcinoma is a very rare disease that accounts for 0.02% of malignant disease in female patients [1]. Most female patients with urethral carcinoma are diagnosed with urothelial carcinoma, squamous cell carcinoma, or adenocarcinoma. We report a significantly rare case of a female patient with urethral adenocarcinoma arising from the Skene duct who presented with serum prostate-specific antigen (PSA) elevation.

## Case presentation

**Fig. 1:**
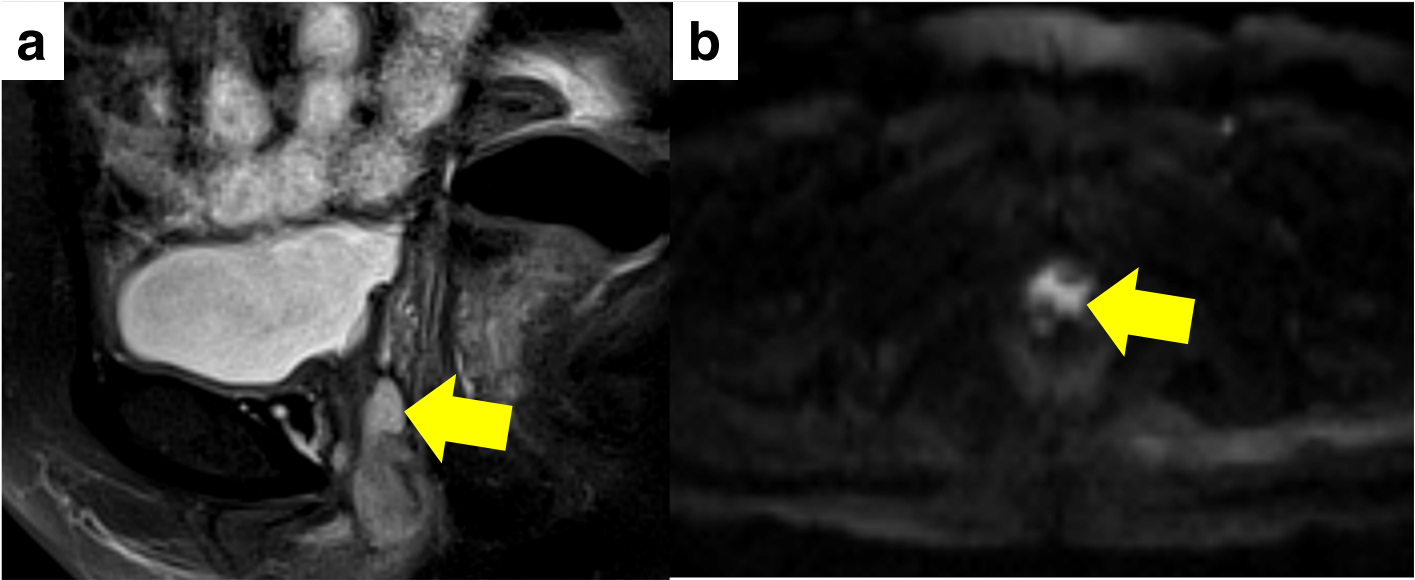
Magnetic resonance images of the urethral tumor (arrows) using (**a**) T2-weighted imaging and (**b**) diffusion-weighted imaging

A 70-year-old Asian Japanese woman was referred to our hospital to undergo further examination for a urethral tumor. She had no remarkable medical history. She had noticed a urethral tumor of 2 cm in diameter 2 years before her initial visit. The tumor was palpable and visible to the eye. She visited a clinician with a chief complaint of a urethral tumor and pain. The tumor, which was located anterior to the vagina wall, had a round surface and a hard nodule. The tumor was located at the distal two-thirds of the urethra. The results of blood and urine examinations were almost within the normal limits, whereas magnetic resonance imaging (MRI) showed an 18 × 10-mm area with high intensity on both T2-weighted imaging and diffusion-weighted imaging studies (Fig. 1). Enhanced computed tomography (CT) showed no lymph node or distant metastasis. Cystourethroscopy revealed no remarkable findings.

A biopsy was performed with the patient under spinal anesthesia to make a pathological diagnosis. The specimens revealed urethral adenocarcinoma with immunohistochemically positive staining for PSA. The patient’s serum PSA level before surgery was 34.4 ng/ml. We recommended total cystectomy with inguinal lymph node resection, but she preferred to preserve her bladder without inguinal lymph node resection owing to concerns about the postoperative complication of edema in her legs. Open total urethral tumor resection was performed with pelvic lymph node resection. We first incised around the ureter with a surgical margin and then changed to a retropubic approach. We then performed cystostomy and lymph node resection. Inguinal lymph node resection was not performed because of the patient’s preference.

**Fig. 2:**
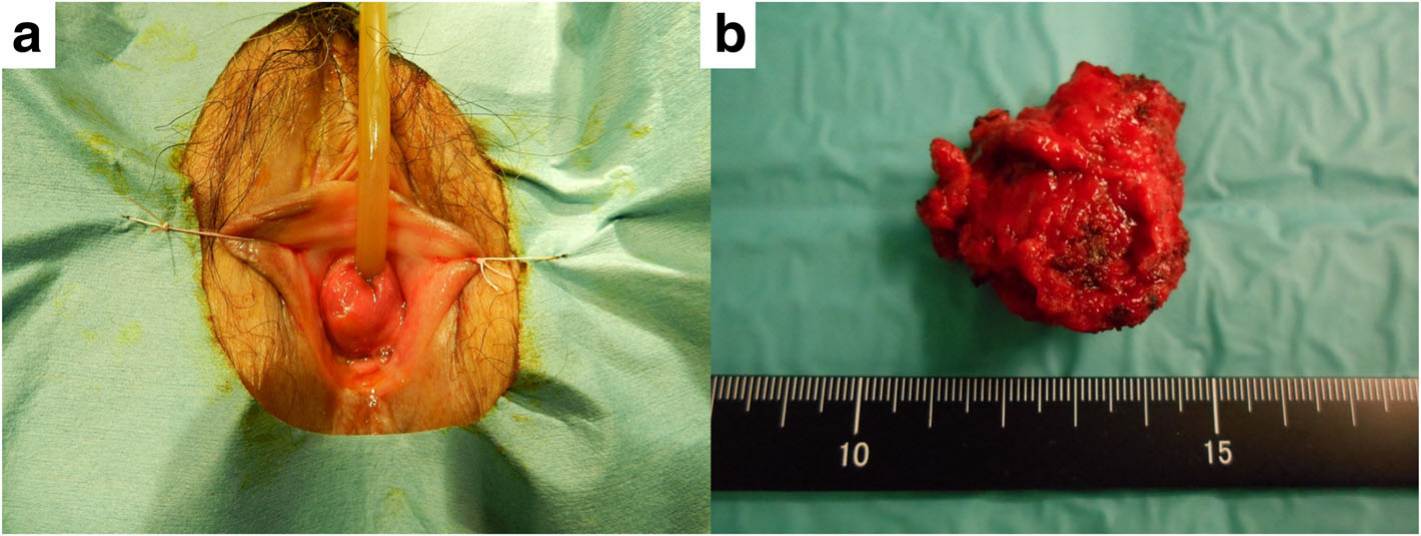
Macroscopic findings of the urethral tumor (**a**) and resected specimens (**b**)

**Fig. 3:**
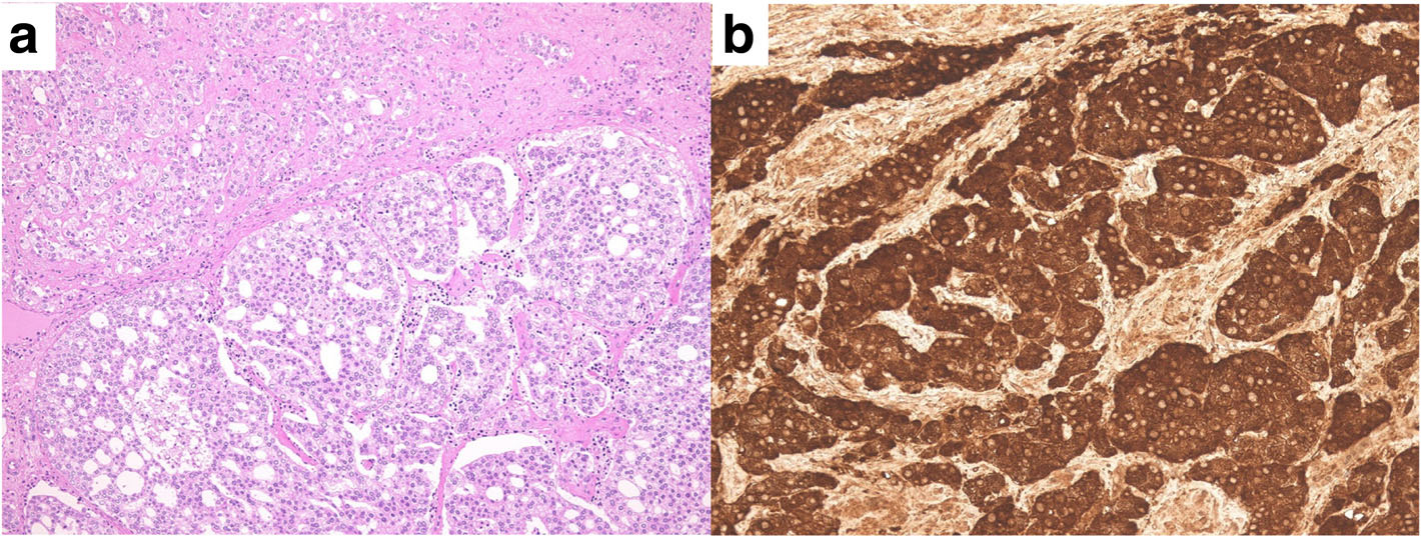
H&E staining (**a**) and prostate-specific antigen staining of urethral tumor (**b**)

The resected tumor was 3.5 × 2.3 × 1.7 cm in size with a smooth, round surface. Macroscopically, the urethral mucosa was intact (Fig. 2). Histologically, the tumor consisted of atypical cells with eosinophilic cytoplasm and enlarged nuclei, forming cribriform, solid, or trabecular structures, which was similar to prostatic cancer. The tumor invaded the vagina. The histological diagnosis was Skene adenocarcinoma based on the results of immunohistochemical staining showing positivity for PSA, P504S, and cancer antigen 125. Cytokeratin 20 and carcinoembryonic antigen were negative (Fig. 3). No metastasis was observed. The final TNM classification was pT3N0M0.

The patient’s serum PSA level decreased to < 0.01 ng/ml after surgery. Adjuvant systemic chemotherapy was not performed. On the basis of CT, the patient has had no recurrence in the 10 months since surgery, and her PSA levels have remained at < 0.01 ng/ml.

## Discussion

Female urethral carcinoma, which accounts for 0.02% of all female malignant disease, usually occurs in individuals who are older than 65 years of age. The disease is roughly divided into the following three types: (1) urothelial cell carcinoma (45–55%), (2) squamous cell carcinoma (19–21%), and (3) adenocarcinoma (16–29%) [1]. The Skene duct is reported to be one of the regions affected by urethral carcinoma. The Skene duct is located in the middle of the urethral smooth muscle, which surrounds the whole urethra; the tissue is thought to be the same as male prostate tissue.

Zaviacic reported that PSA was produced in the female Skene duct, especially on the Skene tissue surface followed by secretion cells, basal cells, and ductal cells [2–4]. Thus, female patients sometimes express serum PSA. Although a few cases of PSA-expressing urethral carcinoma have been reported, our patient’s case represents the first report of a patient with female urethral carcinoma who displayed serum PSA elevation [5]. The chief complaints in patients with female urethral carcinoma are reported to be gross hematuria (62%), palpable tumor (52%), urinary dysfunction (48%), pain (33%), urethrocutaneous fistula (10%), and abscess (5%) [6]. Ultrasound is useful in screening for urethral tumors, then cystoscopy and MRI are performed to detect the detailed tumor location and investigate the extent of invasion [7, 8]. In patients with huge urethral tumors, the tumor becomes enlarged and attains an appearance that is similar to the male prostate [7].

In most cases of nonmetastatic locally advanced female urethral carcinoma, urethral resection is performed with cystostomy or total cystectomy with an ileal conduit. Owing to the low incidence of female urethral carcinoma, there is no confirmed evidence to support the efficacy of inguinal or pelvic lymph node resection in preventing metastasis [9]. Dayyani *et al*. reported the usefulness of cisplatin-based systemic chemotherapy for lymph node metastatic female urethral carcinoma [10]. Other than urothelial carcinoma, no treatments for female urethral carcinoma have been confirmed to be effective. Thus, neoadjuvant systemic chemotherapy is not recommended for locally advanced cases or cases involving local lymph node metastasis. Although there have been no controlled studies to compare the efficacy of surgery and radiotherapy, radiotherapy is reported to be a candidate treatment for localized metastatic and localized advanced urethral carcinoma, especially in patients with urothelial carcinoma and squamous cell carcinoma [11].

The 5-year survival rate in female urethral cancer is reported to be 78% in patients with low-stage urethral cancer and 22% in patients with high-stage urethral cancer. With regard to the tumor location, the 5-year rates in anterior urethral cancer, posterior urethral cancer, and cancer involving the whole urethra are reported to be 54%, 25%, and 18%, respectively. The major site of metastasis is the lung, followed by the liver, bone, and lymph nodes [12].

Urothelial carcinoma is not associated with a particular complaint. Consequently, 66.6% of patients are diagnosed from stage T3 [7]. Owing to the lower incidence of female urothelial carcinoma, there is no established treatment. When female urothelial adenocarcinoma is encountered in patients whose tissue specimens are PSA-positive, the serum PSA level might be a useful tumor marker—even in female patients.

## Conclusions

We describe a very rare case of Skene adenocarcinoma in a female patient with an elevated serum PSA level.
